# Association between vitamin B_6_ status and eczema in children and adolescents: results from the NHANES database

**DOI:** 10.3389/fped.2025.1557186

**Published:** 2025-07-11

**Authors:** Guimei Du, Xinghui Li

**Affiliations:** ^1^ Department of Dermatology, The 906th Hospital of PLA, Ningbo, Zhejiang, China; ^2^ Department of Dermatology, The Yancheng Clinical College of Xuzhou Medical University, The First People’s Hospital of Yancheng, Yancheng, Jiangsu, China

**Keywords:** vitamin B6, 5'-pyridoxal phosphate, 4-pyridoxic acid, childhood eczema, NHANES database, vitamin B6

## Abstract

**Aim:**

There are few studies investigating the relationship between vitamin B6 status and the odds of eczema in children and adolescents. Hereby, this study aims to explore the association between vitamin B6 status and eczema in children and adolescents.

**Methods:**

A cross-sectional study was conducted based on National Health and Nutrition Examination Surveys (NHANES) 2005–2006. The vitamin B6 status was assessed based on levels of 4-PA, PLP and vitamin B6 metabolic rate (4-PA/PLP) by high-performance liquid chromatography. The weighted univariate and multivariate logistics regression models were adopted to explore the association between vitamin B6 status with and the odds of eczema in children and adolescents, with odds ratio (ORs) and 95% confidence intervals (CIs). The subgroup analysis based on age, gender, atopy and body mass index (BMI) were further performed to explore whether the association between vitamin B6 status and eczema in children and adolescents remains robust.

**Results:**

A total of 2,256 eligible children and adolescents were included for further analysis, with the mean aged of 11.81 (±0.09) years old. Among them, 247 (10.95%) had eczema. After adjusted all covariates, we observed high 4-PA was associated with high odds of eczema (OR = 1.57, 95%CI: 1.01–2.44, *P* = 0.044). High 4-PA/PLP was associated with high odds of eczema (OR = 1.46, 95%CI: 1.05–2.03, *P* = 0.028); however, no significant associations were found between dietary vitamin B6 intake and serum PLP level (all *P* > 0.05). The results of subgroup analysis shown that the association between 4-PA and 4-PA/PLP remain robust, especially among children and adolescents aged 6–11 years old, boys, with atopy, and with overweight/obese.

**Conclusion:**

Our study observed that high 4-PA and high vitamin B6 metabolic rate were associated with increased odds of eczema in children and adolescents. Maintaining high vitamin level may have potential benefits in reducing odds for eczema in children and adolescents.

## Introduction

Eczema, featured by defective skin barrier function, is a chronic relapsing skin inflammatory disease worldwide. Approximately, the prevalence of eczema in children has reached 20%, imposing a burden on affected children, families, as well as health care system (1–3). Previous studies reported that the consequences of eczema reach beyond the skin and past childhood and are related to an increased risk of a range of diseases in adulthood, including obese, cardiovascular disease (CVD), and autoimmune disease (4, 5). Hence, positive identification and intervention of the risk factor of childhood eczema is of great significance to reduce the disease burden of childhood eczema to the public health-care systems.

Eczema as an immune disease is closely related to the nutritional changes (6). Vitamin B_6_, including pyridoxine, pyridoxal, and pyridoxamine, is an essential water-soluble vitamin of the B vitamins acting a vital effect in normal brain development and immune system health (7). Previous epidemiological studies suggested that vitamin B6 deficiency is associated with polyneuropathy and dermatitis (8, 9). However, the association between vitamin B6 and skin diseases remains controversial. Miyake et al. (10) reported that after adjustment for confounding factors, there were no evident relationships between vitamin B6 intake and childhood eczema. Therefore, the association between vitamin B6 and childhood eczema needs to be further studied. In the naturally occurring form of vitamin B6, 5'-pyridoxal phosphate (PLP) is the biologically active form of vitamin B6. Meanwhile, 4-pyridoxic acid (4-PA) is the major catabolite of vitamin B6 metabolism. PLP and 4-PA are widely used clinical biomarkers to characterize vitamin B6 levels, which can more accurately reflect the body's vitamin B6 levels than dietary vitamin B6 intake (11). Recently, the ratio of 4-PA to PLP (4-PA/PLP) was considered as a valuable biomarker for assessing vitamin B6 status *in vivo*, and several epidemiologic studies have reported the association between the 4-PA/PLP and some diseases (12–14). Less is known, however, the association between 4-PA, PLP and the ratio of 4-PA to PLP with eczema in children and adolescents.

Herein, based on above background, we explored the association between vitamin B6-related biomarkers with the occurrence of eczema in children and adolescents, aiming to provide data support for the targeted prevention of childhood eczema in the future.

## Methods

### Study design and participants

The study children and adolescents were from the National Health and Nutrition Examination Surveys (NHANES) and data of one survey circle (2005–2006) were extracted. NHANES, a nationally representative survey, aims to assess the nutrient and health status for U.S. civilian population. This database contains personal interviews, laboratory test, and standardized medical examinations (15). The National Health and Nutrition Examination Survey (NHANES) employs a complex, multistage probability sampling design to obtain a nationally representative sample of the U.S. civilian noninstitutionalized population. The survey first selects primary sampling units (counties or groups of contiguous counties), then segments (typically city blocks) within these units, followed by randomly selected households within segments, and finally individuals within households based on demographic characteristics. This design ensures proper representation across age, gender, and racial/ethnic groups. Written informed consent was obtained from all participants, and the survey protocol was approved by the National Center for Health Statistics (NCHS) Research Ethics Review Board.

In present study, 2,849 children and adolescents aged 6–17 years old were initially included. Among them, 262 children missing dietary vitamin B6 intake information, 323 children and adolescents missing data of PLP and 4-PA and 8 children and adolescents missing diagnostic information of eczema were further excluded. Finally, 2,256 eligible children and adolescents were included for final analysis.

### Diagnosis of eczema in children and adolescents

The diagnosis of eczema in children and adolescents was based on the self-reported NHANES questionnaire. Children and adolescents answered “yes” to “Has a doctor or other health professional ever told you that you have eczema?” were diagnosed with eczema (16). For children aged 6–8 years old, the questionnaires were responded by their guardian; children aged 9–11 years old were accompanied by their guardian to assist in responding, while adolescents aged 12–17 years old were responded by themselves.

### Assessment of dietary vitamin B_6_ intake

The data of dietary vitamin B6 were obtained by the 24-h dietary recall interview in the NHANES. This recall interview requires subjects to report all food and beverages consumed prior 24-h to the interview. Dietary consumption data were converted to the United States Department of Agriculture (USDA) standard reference codes, and dietary intake were linked to the USDA's Food and Nutrient Database for Dietary Studies (FNDDS) (17). In present study, the vitamin B6 intake were categorized to three levels according to its tertiles: <1.18, 1.18–1.97 and ≥1.97 mg.

### Measurement serum PLP and 4-Pa concentrations

The serum PLP (nmol/L) and 4-PA (nmol/L), as well as their ratio 4-PA/PLP were used to evaluate vitamin B_6_ status. The 4-PA and PLP were determined using high-performance liquid chromatography (HPLC). Further detailed laboratory measurement procedures and quality control can be accessed at the Mobile Examination Center on the NHANES website (17). The higher the vitamin B6 metabolic rate expressed as the 4-PA/PLP value, the lower the vitamin B6 level in the body (18).

### Potential covariates

In present study, we extracted social demographic information [age, gender, race, poverty-to-income ratio (PIR) and household education level], lifestyle (physical activity, screen time and tobacco exposure) complications (atopy, asthma, and hay fever), physical examination [body mass index (BMI) and birth weight], and laboratory parameters [4-PA, PLP, CRP (c-reactive protein)].

The physical activity (yes/no) of children aged 6–11 years old was assessed by the question “How many times per week does your play or exercise enough to make him/her sweat and breathe hard?” and more than one time was defined as “yes”; for adolescents aged 12–17 years old, the “yes” to “Over the past 30 day you walked or bicycled as part of getting to and from work, or school, or to do errands?” was defined as doing physical activity. Screen time was calculated based on the question “Over the past 30 days, on average about how many hours per day did you sit and watch TV, videos, use a computer or play computer games?” (< 3 h/≥3 h). The overall NHANES 2005–2006 Allergy Component is designed to assess the allergen exposure, allergic sensitization, allergic symptoms and diseases, and their complex relationship in the general U.S. population. Accordingly, in present study, atopy was defined as a binary variable indicating serum levels ≥0.35 kU/L for any of the 19 specific IgE antibodies measured (LBXID2, LBXID1, LBXIE1, LBXIE5, LBXII6, LBXIM6, LBXF13, LBXIF1, LBXIF2, LBXIW1, LBXIG5, LBXIG2, LBXIT7, LBXIT3, LBXF24, LBXIM3, LBXW11, LBXE72, LBXE74). The complete documentation of these IgE codes, including their corresponding allergens and detection methodologies, is available in the official NHANES laboratory manual: https://wwwn.cdc.gov/Nchs/Data/Nhanes/Public/2005/DataFiles/AL_IGE_D.htm#LBXID1. Children and adolescents who answered “yes” to the question “Has a doctor or other health professional ever told you that you had asthma?” were defined as having asthma history. Birth weight was categorized into four groups: <5.5, 5.5–8.9, ≥ 9 and unknown (19). Tobacco exposure was assessed by the question “Does anyone smoke in the home?” (yes/no). BMI was converted to a BMI Z-score accounting for age and gender using recommended CDC percentiles. A BMI Z-score of ≥85th percentile and <95th percentile indicates overweight status, and a BMI Z-score of ≥95th percentile indicates obesity (20).

### Statistical analysis

Continuous data were expressed as mean and standard error (S.E.), and the weighted t-test was used for comparison between groups. Categorical variables were described as the number and percentage [*N* (%)], and comparisons between groups used the weighted chi-square test or Fisher's test. The univariate logistics regression analysis was conducted to screen the covariates related to eczema in children and adolescents (Supplementary Table S1). The weighted multivariate logistics regression analysis was utilized to evaluate the association between vitamin B_6_ status and eczema, with odds ratio (ORs) and 95% confidence intervals (CIs). Multivariate imputation by chained equations (MICE) was used to missing data imputation. Sensitivity analysis was performed before and after missing data imputation (Supplementary Table S2). Model 1 adjusted age, gender and race; model 2 adjusted age, race, asthma history, hay fever and birth weight. Subgroups analysis based on different age, gender, atopy and BMI were further performed to evaluate whether the association between vitamin B_6_ status and eczema remain robust. All statistical analyzes were performed using R v 4.20 (R Foundation for Statistical Computing, Vienna, Austria) and SAS v 9.4 (SAS Institute, Cary, North Carolina) software. Two-sided *P*-value <0.05 was considered statistically significant.

## Results

### Characteristics of study population

**Figure 1:**
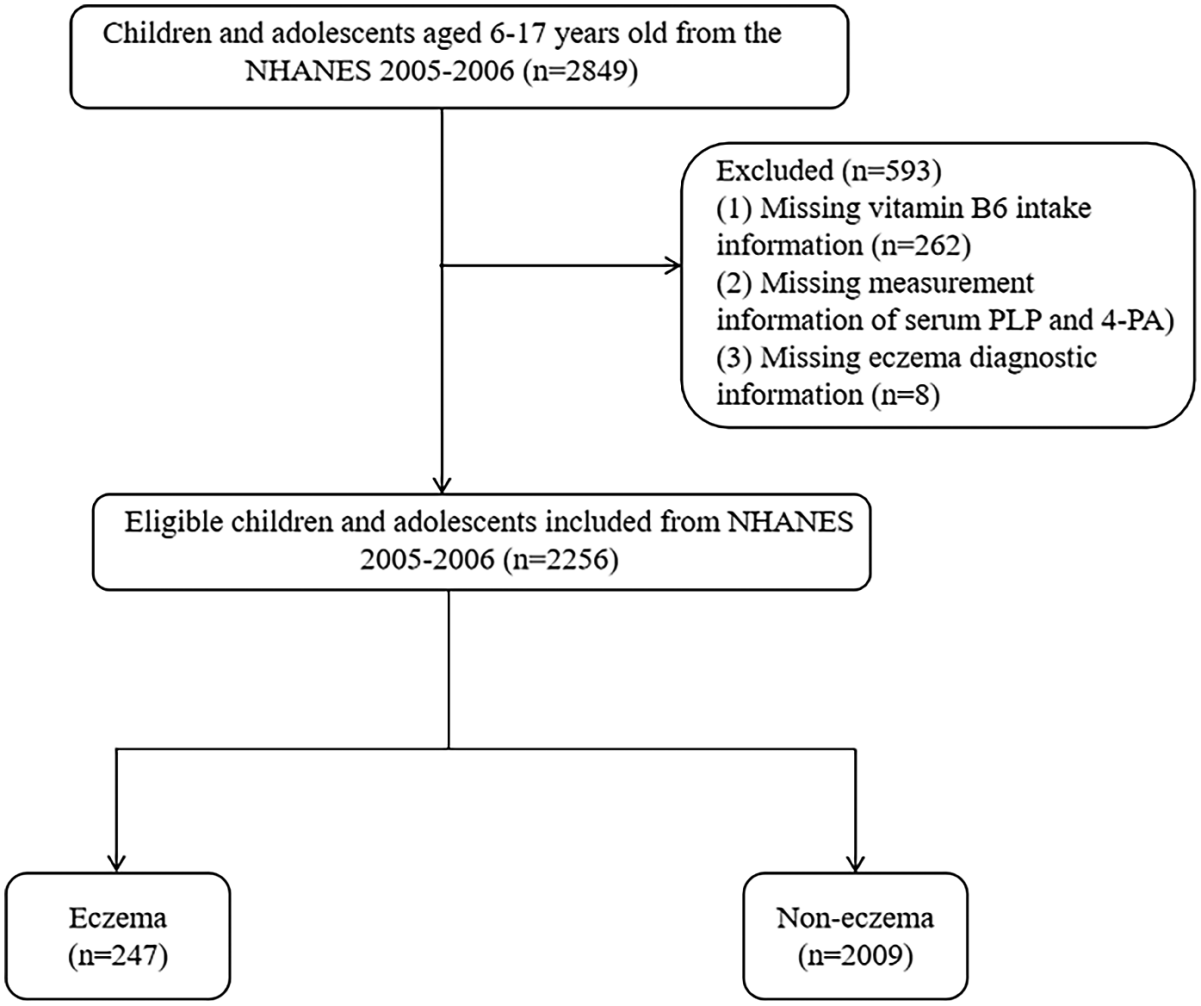
Participant selection flowchart for the analysis of vitamin B6 status and eczema association in US children (NHANES 2005–2006). Note: NHANES, National Health and Nutrition Examination Surveys; PLP, pyridoxal 5'-phosphate; 4-PA, 4-pyridoxic acid.

The flow chart of population screening was shown in Figure 1. Finally, 2,256 eligible children and adolescents were included, with the mean age of 11.81 (±0.09) years old. Among them, 247 (10.95%) had eczema. The level of 4-PA in children and adolescents with eczema was significant higher than in children and adolescents without eczema [32.55 (±1.61) nmol/L *vs.* 27.88 (±1.32) nmol/L)]. Difference was found between age, race, the history of asthma and hay fever, the level of birth weight and 4-PA between eczema and non-eczema groups (all *P* < 0.05). Characteristics of included children and adolescents were shown in Table 1.

**Table 1:** Characteristics of children and adolescents.

Variable	Total (*n* = 2,256)	Eczema (*n* = 247)	Non-eczema (*n* = 2,009)	*P*
Age, years, mean (S.E)	11.81 (0.09)	10.96 (0.27)	11.94 (0.10)	0.004
Gender, *n* (%)				0.651
Female	1,138 (48.83)	130 (50.89)	1,008 (48.52)	
Male	1,118 (51.17)	117 (49.11)	1,001 (51.48)	
Race/ethnicity, *n* (%)				<0.001
Non-Hispanic White	614 (62.60)	82 (67.11)	532 (61.93)	
Non-Hispanic Black	700 (13.62)	115 (18.14)	585 (12.95)	
Mexican American	759 (12.66)	27 (3.54)	732 (14.00)	
Other race	183 (11.13)	23 (11.21)	160 (11.11)	
PIR, *n* (%)				0.081
≤3.5	1,724 (64.32)	167 (57.37)	1,557 (65.34)	
>3.5	532 (35.68)	80 (42.63)	452 (34.66)	
Household education level, *n* (%)				0.076
Less than 9th Grade	278 (5.90)	9 (3.57)	269 (6.24)	
9–11th Grade	428 (12.52)	40 (12.05)	388 (12.59)	
High school grad/GED or equivalent	532 (25.29)	58 (22.04)	474 (25.77)	
Some college or AA degree	620 (31.67)	74 (29.18)	546 (32.04)	
College graduate or above	398 (24.63)	66 (33.16)	332 (23.37)	
Physical activity, *n* (%)				0.629
No	82 (2.49)	6 (2.01)	76 (2.56)	
Yes	2,174 (97.51)	241 (97.99)	1,933 (97.44)	
Screen time, *n* (%)				0.590
<3 h	757 (41.04)	83 (42.47)	674 (40.83)	
≥3 h	1,499 (58.96)	164 (57.53)	1,335 (59.17)	
Atopy, *n* (%)				0.060
No	1,077 (53.78)	95 (47.19)	982 (54.76)	
Yes	1,179 (46.22)	152 (52.81)	1,027 (45.24)	
Asthma, *n* (%)				<0.001
No	1,880 (82.42)	171 (71.14)	1,709 (84.08)	
Yes	376 (17.58)	76 (28.86)	300 (15.92)	
Hay fever, *n* (%)				<0.001
No	2,168 (95.76)	222 (89.83)	1,946 (96.63)	
Yes	88 (4.24)	25 (10.17)	63 (3.37)	
Birth weight, pounds, *n* (%)				0.002
<5.5	205 (8.46)	31 (10.77)	174 (8.11)	
5.5–8.9	1,338 (62.78)	163 (72.32)	1,175 (61.37)	
≥9	152 (7.14)	10 (3.54)	142 (7.67)	
Unknown	561 (21.62)	43 (13.37)	518 (22.84)	
BMI, *n* (%)				0.513
Normal	1,368 (65.54)	154 (65.67)	1,214 (65.51)	
Overweight	364 (15.54)	50 (17.56)	314 (15.25)	
Obesity	524 (18.92)	43 (16.76)	481 (19.24)	
Vitamin B_6_ intake, mg, Mean (S.E)	1.80 (0.05)	1.66 (0.13)	1.82 (0.05)	0.187
Vitamin B_6_ intake, mg, *n* (%)				0.125
<1.18	751 (32.93)	96 (39.59)	655 (31.95)	
1.18–1.94	742 (33.96)	86 (32.82)	656 (34.13)	
≥1.94	763 (33.11)	65 (27.59)	698 (33.92)	
Energy intake, kcal, mean (S.E)	2,182.21 (34.85)	2,117.27 (78.25)	2,191.80 (35.85)	0.359
4-PA, nmol/L, mean (S.E)	28.48 (1.19)	32.55 (1.61)	27.88 (1.32)	0.041
4-PA, nmol/L, *n* (%)				0.059
<16.26	1,018 (32.84)	93 (25.34)	925 (33.95)	
16.26–28.49	679 (34.05)	83 (36.98)	596 (33.61)	
≥28.49	559 (33.11)	71 (37.68)	488 (32.44)	
PLP, nmol/L, mean (S.E)	64.60 (1.72)	69.20 (4.12)	63.92 (1.58)	0.168
PLP, nmol/L, *n* (%)				0.882
<41.69	865 (33.00)	95 (32.34)	770 (33.10)	
41.69–68.80	765 (33.69)	83 (32.75)	682 (33.83)	
≥68.80	626 (33.31)	69 (34.91)	557 (33.08)	
Ratio of 4-PA to PLP, mean (S.E)	0.46 (0.01)	0.50 (0.02)	0.46 (0.01)	0.099
Ratio of 4-PA to PLP *n* (%)				0.272
<0.33	887 (32.97)	84 (28.51)	803 (33.63)	
0.33–0.48	770 (33.99)	88 (34.08)	682 (33.97)	
≥0.48	599 (33.04)	75 (37.40)	524 (32.40)	
CRP, mg/dl, mean (S.E)	169.52 (11.72)	199.51 (21.19)	165.08 (14.23)	0.255
Tobacco exposure, *n* (%)				0.194
No	1,866 (83.17)	201 (85.71)	1,665 (82.79)	
Yes	390 (16.83)	46 (14.29)	344 (17.21)	

S.E, standard error; PIR, poverty-to-income ratio; PIR ≤3.5, medium and low income; PIR >3.5, high income; GED, general equivalent diploma; BMI, body mass index; 4-PA, 4-pyridoxic acid; PLP, pyridoxal 5'-phosphate; CRP, C-reactive protein.

### Association between vitamin B6 status and eczema

We employed two weighted multivariate logistics regression models to evaluate the association between dietary vitamin B6, 4-PA, PLP, and 4-PA/PLP with eczema, as depicted in Table 2. In fully adjusted model 2, no significant associations were observed between dietary vitamin B_6_ intake and PLP levels and the odds of eczema (*P* > 0.05). Compare to children and adolescents with low and medium 4-PA levels, those with high 4-PA levels had a high odds of eczema (OR = 1.57, 95%CI: 1.01–2.44, *P* = 0.044). Moreover, the results also shown that a high ratio of 4-PA to PLP was associated with a high odd of eczema (OR = 1.46, 95%CI: 1.05–2.03, *P* = 0.028).

**Table 2:** Correlations between biomarkers of vitamin B_6_ status and eczema in children and adolescents.

Variables	Model 1		Model 2	
	OR (95%CI)	*P*	OR (95%CI)	*P*
Vitamin B_6_ intake, mg				
<1.18	Ref		Ref	
1.18–1.97	0.77 (0.51–1.15)	0.185	0.76 (0.51–1.15)	0.182
≥1.97	0.71 (0.42–1.19)	0.177	0.70 (0.45–1.09)	0.110
4-PA, nmol/L				
<16.22	Ref		Ref	
16.22–26.65	1.61 (1.04–2.51)	0.036	1.55 (0.98–2.45)	0.057
≥26.66	1.67 (1.07–2.60)	0.027	1.57 (1.01–2.44)	0.044
PLP, nmol/L				
<41.69	Ref		Ref	
41.69–68.86	0.96 (0.59–1.55)	0.847	0.93 (0.58–1.50)	0.761
≥68.87	1.00 (0.58–1.75)	0.991	0.97 (0.59–1.59)	0.899
Ratio of 4-PA to PLP				
<0.33	Ref		Ref	
0.33–0.48	1.28 (0.77–2.15)	0.318	1.22 (0.72–2.09)	0.431
≥0.48	1.56 (1.12–2.19)	0.013	1.46 (1.05–2.03)	0.028

OR, odd ratio; CI, confidence interval; Ref, reference; 4-PA, 4-pyridoxic acid; PLP, pyridoxal-5′-Phosphate; Model 1 was adjusted for age, gender, race/ethnicity; Model 2 was additionally adjusted for asthma, hay fever and birth weight.

### Subgroup analysis based on different age, gender, atopy and BMI

**Figure 2:**
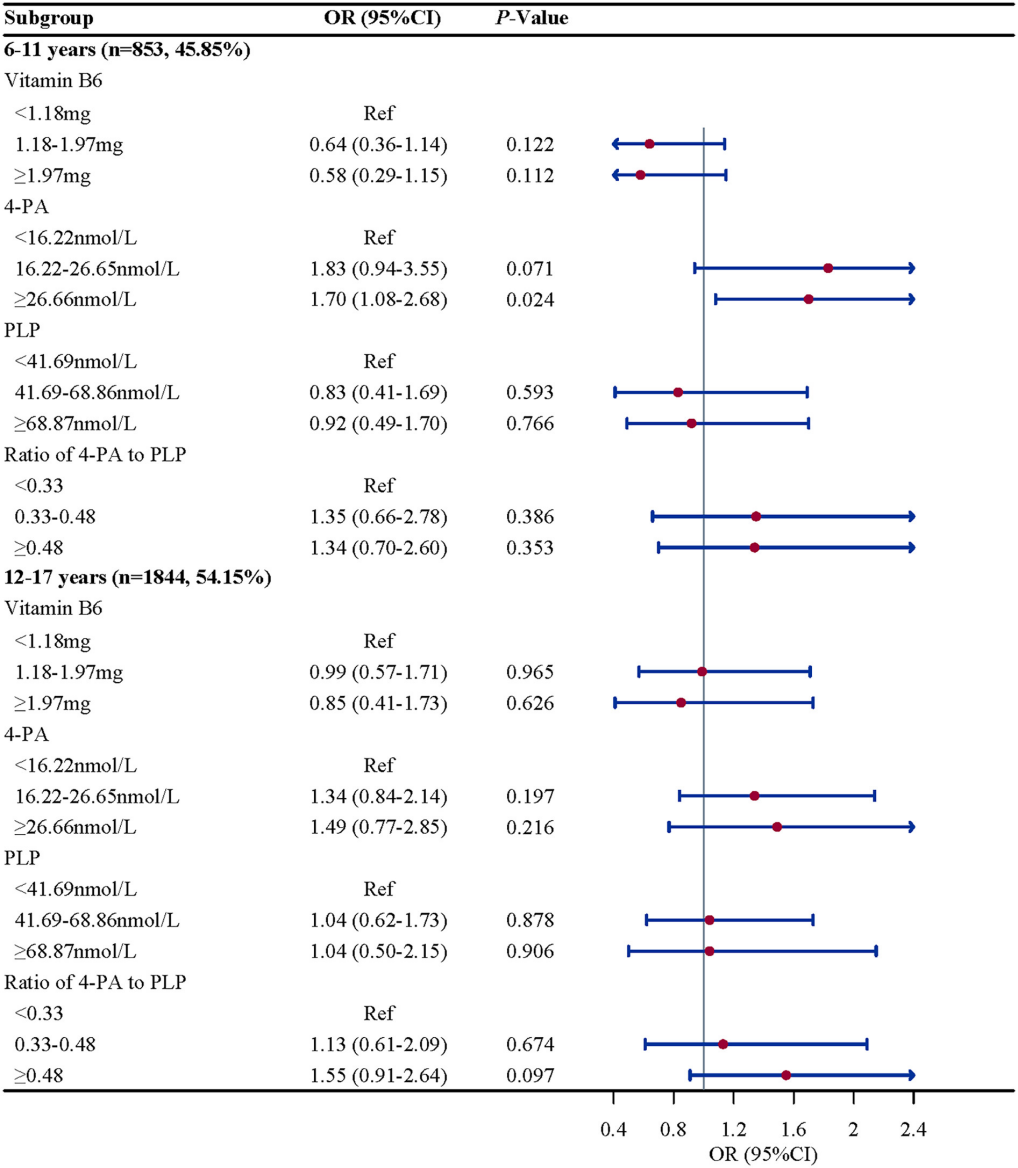
Age-stratified associations of vitamin B6 status with eczema in US children and adolescents (NHANES 2005–2006). Note: OR, odds ratio; CI, confidence intervals; PLP, pyridoxal 5'-phosphate; 4-PA, 4-pyridoxic acid.

**Figure 3:**
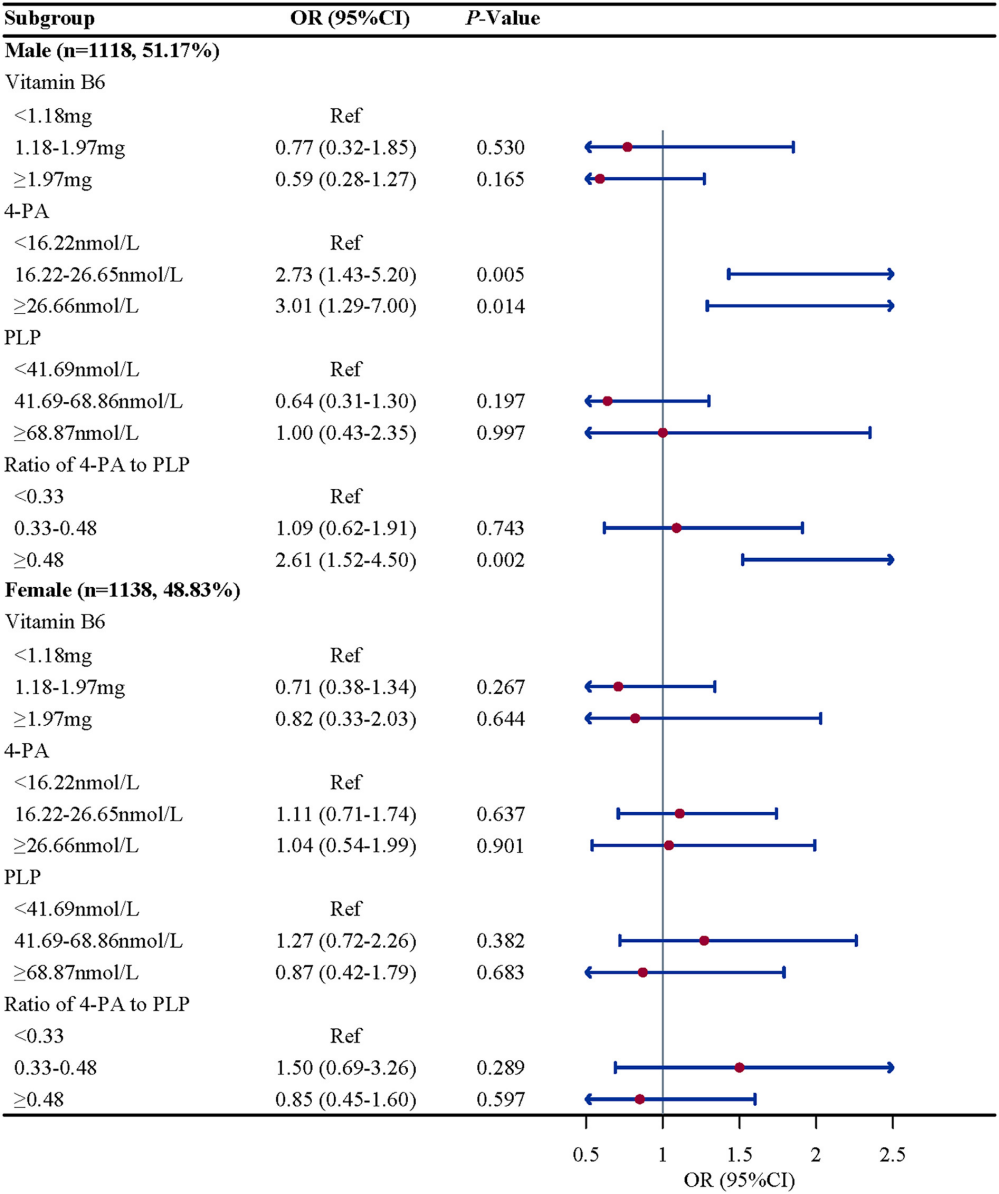
Gender-stratified associations between vitamin B6 status and eczema in US children and adolescents (NHANES 2005–2006). Note: OR, odds ratio; CI, confidence intervals; PLP, pyridoxal 5'-phosphate; 4-PA, 4-pyridoxic acid.

**Figure 4:**
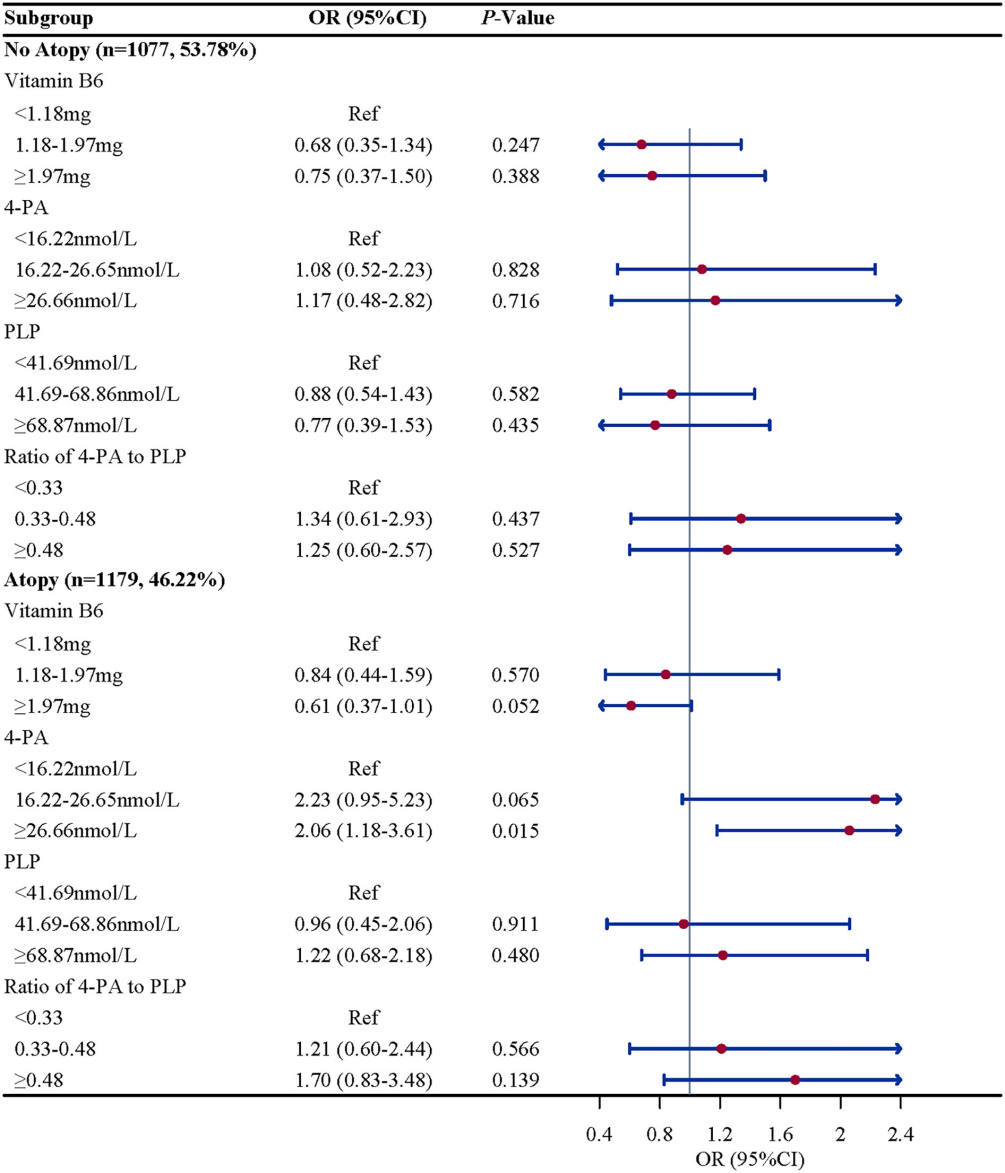
Atopy history-stratified associations between vitamin B6 status and eczema in US children and adolescents (NHANES 2005–2006). Note: OR, odds ratio; CI, confidence intervals; PLP, pyridoxal 5'-phosphate; 4-PA, 4-pyridoxic acid.

**Figure 5:**
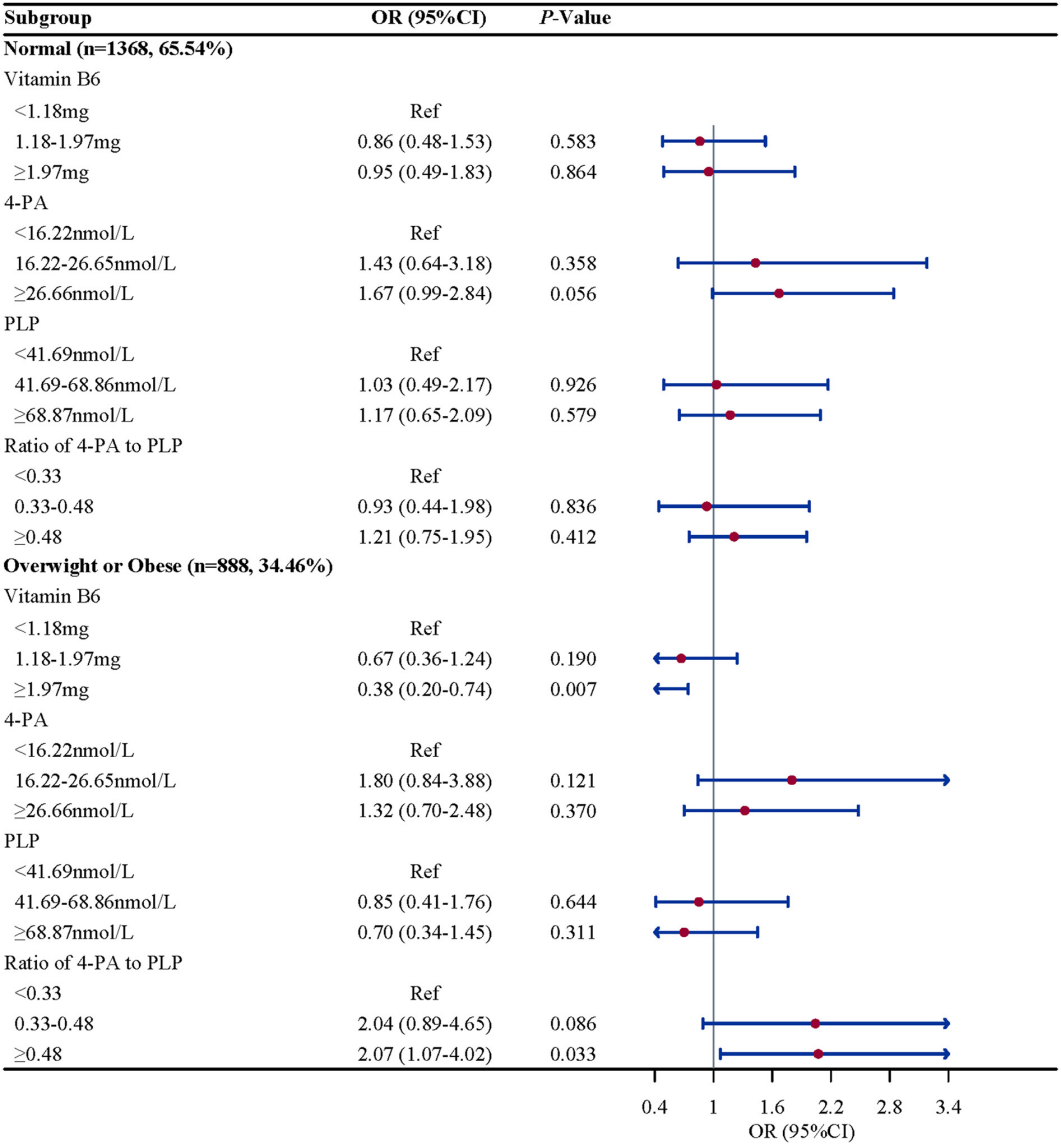
BMI-stratified associations between vitamin B6 status and eczema in US children and adolescents (NHANES 2005–2006). Note: BMI, body mass index.

Subgroup analysis were performed to further evaluate whether the association between vitamin B6 status and eczema remain robust. When stratified by gender and BMI, we found the association between the ratio of 4-PA to PLP and eczema remain robust, especially in boys (OR = 2.61, 95%CI: 1.52–4.50, *P* = 0.002) and children and adolescents with overweight/obese (OR = 2.07, 95%CI: 1.07–4.02, *P* = 0.033). When stratified by age and atopy, we found the association between 4-PA and eczema also remain robust, especially in children and adolescents aged 6–11 years (OR = 1.70, 95%CI: 1.08–2.68, *P* = 0.112) and with atopy (OR = 2.06, 95%CI: 1.18–3.61, *P* = 0.015) (Figures 2–5).

## Discussion

Based on the findings of NHANES database, we found positive correlation between serum 4-PA levels and 4-PA/PLP with the odds of eczema in children and adolescents. No significant associations were found between dietary B6 intake and serum PLP with the odds of eczema in this population. These associations remain robust after stratified analysis based on age, gender, atopy and BMI. To our best knowledge, this was the first cross-sectional to explore the association between biomarkers of vitamin B6 and eczema in children and adolescents in the United States.

Primary atopic diseases (PAD) are monogenic inherited diseases characterized by allergic or atopic-related symptoms. Atopic dermatitis, usually eczema, is one of the skin lesions characteristic of PAD. More and more researchers have found that disorders of vitamin levels are related to skin conditions, and systemic and topic treatments have shown significant improvements. Vitamin B6 is a water-soluble vitamin that can be obtained from a variety of foods and has been shown to be closely linked to inflammation and immunity levels in the body (21). A cross-sectional from the Korean Child Health and Environment enrolled 2,333 children aged 6–8 years to investigate the association between dietary methyl donor intake with asthma and atopy (22). Dietary methyl donors participate in homocysteine ​​metabolism, providing methyl groups to the body and thus maintaining the balance of methyl reaction. Vitamin B6 has been shown to be an important dietary methyl donor (23). That study found high vitamin B6 intake were associated with the reduced risk of atopy as well as asthma symptoms. A small cohort study from rural southwestern Sweden investigated the association between prenatal diet and allergic disorders, including childhood eczema. The authors reported that vitamin B6 metabolism was significantly associated with the risk of future allergic disease (24). However, the association between vitamin B6 and atopy diseases remain clinically controversial. The Osaka Maternal and Child Health Study prospectively explored the association between maternal vitamin B intake during pregnancy and infant wheeze and eczema and reported that after adjusted several confounding, there was no significant evidence to support the association between vitamin B6 intake and the risk of wheeze or eczema in the offspring (10). These studies have reported inconsistent associations between vitamin B6 and childhood eczema, which can partly attribute to differences of ethnic groups, outcomes and exposures definitions.

Currently, there is controversy about the association between vitamin B6 and childhood eczema due to the research population, definition of disease and exposure factors. Therefore, more evidence is needed to further explore the association between the vitamin B6 intake and childhood eczema. Our study recruited 2,256 children and adolescents aged 6–17 years from the NHANES database in 2005–2006, and used vitamin B6 metabolites as exposure to explore the association between vitamin B6 and childhood eczema. After considering a series of confounding factors that affect childhood eczema, we observed a positive association between 4-PA and childhood eczema. No significant association was observed between PLP and childhood eczema, and there was a positive correlation between the vitamin B6 metabolic rate represented by 4-PA/PLP and childhood eczema. Considering children's age, gender, BMI, and the history of atopy diseases may affect the outcomes, we performed subgroup analysis subsequently to verify whether the association between vitamin B6 levels and childhood eczema remained robust in different subgroups. The association between vitamin B6 levels and childhood eczema remain robust, especially in children aged 6–11years, boys, with the history of atopy diseases and with the overweight/obesity. Our study provides strong evidence for a beneficial association between high vitamin B6 levels and eczema in children. Several physiological mechanisms could support and explain these findings. From the perspective of inflammation, vitamin B6 has antioxidant and anti-inflammatory biological functions due to its γ-hydroxyl group on the pyridine structure, which can effectively quench singlet oxygen (25). Previous studies have shown that a lack of vitamin B6 can lead to a decrease in the body's antioxidant defense ability (26, 27). Inflammation is thought to result from disruption of the epidermal barrier and activation of epidermal inflammatory dendrites and innate lymphoid cells, which attract and interact with Th2 cells. The direct mechanism of eczematous lesions is inflammation associated with Th2 cell dysregulation. Activated T cells release cytokines into the skin, primarily interleukin-4, interleukin-13, and interleukin-31, which activate the downstream Janus kinase (JAK) pathway (28). Moreover, vitamin B6 is also associated with an imbalance in the microbial levels of the intestinal flora, which mediates autism-like behaviors by regulating the metabolism of vitamin B6 (29). The pathogenesis of eczema involves a complex interaction between defective epidermal barriers and microbial imbalance, leading to allergen penetration and stimulation of type 2 helper T cell responses (30). Alterations in the microbiome have been shown to contribute to susceptibility and exacerbation of eczema. Previous studies have demonstrated that children with eczema have reduced gut microbial diversity compared with healthy children (31).

Previous researches have focused on the relationship between vitamin B6 and chronic diseases in disease-specific populations, while evidence from studies in child and adolescent populations is limited. We utilized a large, high-quality NHANES dataset and performed comprehensive correction for confounding factors including age, race, asthma, hay fever and birth weight that affect childhood eczema to produce robust results. Moreover, our study used serum PLP and 4-PA measurements that better reflect bioavailability compared with dietary questionnaires. Our findings carry important clinical implications for pediatric eczema management. The association between PLP levels and eczema suggests that vitamin B6 status assessment could be incorporated into routine nutritional screening for children with eczema, particularly in those with atopic predisposition. Clinicians may consider evaluating plasma PLP levels when standard therapies show limited efficacy, as suboptimal B6 status might exacerbate inflammatory pathways. For patients, these results highlight the potential dual benefit of maintaining adequate vitamin B6 status—not only supporting basic metabolic functions but possibly modulating eczema severity. This could be achieved through dietary counseling focusing on B6-rich foods (e.g., poultry, fish, bananas) or targeted supplementation in deficient cases, though clinical trials are needed to confirm therapeutic efficacy. Our study still has several limitations that warrant attention. First, due to the cross-sectional nature of the study, only preliminary estimates of the association between vitamin B6-related biomarkers and childhood eczema can be made, and we cannot conclude the cause effect of this association. Second, although we adjusted for as many confounders as possible for childhood eczema, we were unable to exclude the influence of potential confounders on the outcomes. Thirdly, several covariates information was obtained through the NHANES questionnaire, which may be subject to recall bias. Finally, as the NHANES database covers a representative U.S. population, extrapolating these findings to other populations requires caution. Future studies should prioritize longitudinal assessments to establish temporal relationships between B6 status and eczema. Additionally, randomized controlled trials of B6 supplementation in pediatric eczema patients stratified by baseline nutritional status are warranted. Finally, fundamental experimental research remains necessary to elucidate the mechanistic role of vitamin B6in skin microbiome modulation.

## Conclusion

Among the study children and adolescents in present study, we observed a positive association between serum 4-PA and the odds of eczema, and a positive association between the metabolic rate of vitamin B6 and the odds of eczema. These findings suggest that maintaining a higher vitamin B6 level may have a potential benefit in preventing eczema in this population.

## Data Availability

Publicly available datasets were analyzed in this study. This data can be found here: NHANES database, https://wwwn.cdc.gov/nchs/nhanes/.
