# Supplementary material for: Association between vitamin B6 status and eczema in children and adolescents: results from the NHANES database
Source: Front Pediatr. 2025 Jul 11;13:1557186. doi: 10.3389/fped.2025.1557186 (PMC12289665; doi:10.3389/fped.2025.1557186)
Supplement: Supplementary file 1 [file Table1.docx]

Table S1 Sensitivity analyses before and after imputation of missing data

| Variables | OR (95%CI) | *P* |
| --- | --- | --- |
| Vitamin B_6_, mg |  |  |
| <1.18 | Ref |  |
| 1.18-1.97 | 0.77 (0.51-1.16) | 0.195 |
| ≥1.97 | 0.68 (0.44-1.05) | 0.079 |
| 4-PA, nmol/L |  |  |
| <16.22 | Ref |  |
| 16.22-26.65 | 1.52 (0.94-2.45) | 0.085 |
| ≥26.66 | 1.57 (1.01-2.44) | 0.044 |
| PLP, nmol/L |  |  |
| <41.69 | Ref |  |
| 41.69-68.86 | 0.91 (0.57-1.46) | 0.671 |
| ≥68.87 | 0.97 (0.59-1.58) | 0.884 |
| Ratio of 4-PA to PLP |  |  |
| <0.33 | Ref |  |
| 0.33-0.48 | 1.21 (0.71-2.05) | 0.466 |
| ≥0.48 | 1.47 (1.06-2.04) | 0.025 |

OR: odd ratio; CI: confidence interval; Ref: reference;
4-PA: 4-pyridoxic acid; PLP: pyridoxal-5′-Phosphate;
Model 1 was adjusted for age, gender, race/ethnicity;
Model 2 was additionally adjusted for asthma, hay fever and birth weight.

Table S2 Univariate logistic regression analysis of eczema

| Variable | OR (95%CI) | *P* |
| --- | --- | --- |
| Age | 0.92 (0.87-0.97) | 0.005 |
| Gender |  |  |
| Female | Ref |  |
| Male | 0.91 (0.58-1.42) | 0.659 |
| Race/Ethnicity |  |  |
| Non-Hispanic White | Ref |  |
| Non-Hispanic Black | 1.29 (0.96-1.74) | 0.088 |
| Mexican American | 0.23 (0.14-0.39) | <0.001 |
| Other Race | 0.93 (0.41-2.11) | 0.854 |
| PIR |  |  |
| ≤3.5 | Ref |  |
| >3.5 | 1.40 (0.91-2.15) | 0.113 |
| HH Ref Person Education Level |  |  |
| Less Than 9th Grade | Ref |  |
| 9-11th Grade | 1.68 (0.52-5.35) | 0.358 |
| High School Grad/GED or equivalent | 1.50 (0.47-4.81) | 0.474 |
| Some College or AA degree | 1.59 (0.56-4.51) | 0.355 |
| College Graduate or above | 2.48 (0.91-6.80) | 0.074 |
| Physical activity |  |  |
| No | Ref |  |
| Yes | 1.28 (0.43-3.84) | 0.639 |
| Screen time |  |  |
| <3 h | Ref |  |
| ≥3 h | 0.93 (0.71-1.22) | 0.600 |
| Atopy |  |  |
| No | Ref |  |
| Yes | 1.35 (0.96-1.91) | 0.078 |
| Asthma |  |  |
| No | Ref |  |
| Yes | 2.14 (1.45-3.18) | <0.001 |
| Hay fever |  |  |
| No | Ref |  |
| Yes | 3.25 (1.56-6.79) | 0.004 |
| Birth weight |  |  |
| <5.5 pounds | Ref |  |
| 5.5-8.9 pounds | 0.89 (0.45-1.76) | 0.716 |
| ≥9 pounds | 0.35 (0.09-1.29) | 0.106 |
| Unknown | 0.44 (0.22-0.90) | 0.026 |
| BMI |  |  |
| Normal | Ref |  |
| Overweight | 1.15 (0.71-1.86) | 0.549 |
| Obesity | 0.87 (0.60-1.26) | 0.435 |
| Energy intake | 1.00 (1.00-1.00) | 0.388 |
| CRP | 1.08 (0.80-1.46) | 0.573 |
| Tobacco exposure |  |  |
| No | Ref |  |
| Yes | 0.80 (0.55-1.16) | 0.225 |

OR: odd ratio; CI: confidence interval; Ref: reference; 4-PA: 4-pyridoxic acid; PLP: pyridoxal-5′-Phosphate; PIR: poverty-to-income ratio; BMI: body mass index; CRP: c-reactive protein.
